# A quantitative reverse-transcriptase PCR assay for the assessment of drug activities against intracellular *Theileria annulata* schizonts

**DOI:** 10.1016/j.ijpddr.2014.09.003

**Published:** 2014-09-19

**Authors:** Isabel Hostettler, Joachim Müller, Chad E. Stephens, Richard Haynes, Andrew Hemphill

**Affiliations:** a Institute for Parasitology, Vetsuisse Faculty, University of Berne, Länggass-Strasse 122, 3012 Bern, Switzerland; b Department of Chemistry and Physics, Augusta State University, Augusta, GA 30904-2200, USA; c Department of Chemistry, Open Laboratory of Chemical Biology, Institute of Molecular Technology for Drug Discovery and Synthesis, The Hong Kong University of Science and Technology, Clear Water Bay, Kowloon, Hong Kong Special Administrative Region

**Keywords:** *Theileria*, Theileriosis, Apicomplexa, Chemotherapy, Real time PCR, Electron microscopy, Apoptosis

## Abstract

Intracellular schizonts of the apicomplexans *Theileria annulata* and *Theileria parva* immortalize bovine leucocytes thereby causing fatal immunoproliferative diseases. Buparvaquone, a hydroxynaphthoquinone related to parvaquone, is the only drug available against *Theileria*. The drug is only effective at the onset of infection and emerging resistance underlines the need for identifying alternative compounds. Current drug assays employ monitoring of proliferation of infected cells, with apoptosis of the infected host cell as a read-out, but it is often unclear whether active compounds directly impair the viability of the parasite or primarily induce host cell death. We here report on the development of a quantitative reverse transcriptase real time PCR method based on two *Theileria* genes, *tasp* and *tap104*, which are both expressed in schizonts. Upon *in vitro* treatment of *T. annulata* infected bovine monocytes with buparvaquone, TaSP and Tap104 mRNA expression levels significantly decreased in relation to host cell actin already within 4 h of drug exposure, while significant differences in host cell proliferation were detectable only after 48–72 h. TEM revealed marked alterations of the schizont ultrastructure already after 2 h of buparvaquone treatment, while the host cell remained unaffected. Expression of TaSP and Tap104 proteins showed a marked decrease only after 24 h. Therefore, the analysis of expression levels of mRNA coding for TaSP and Tap104 allows to directly measuring impairment of parasite viability. We subsequently applied this method using a series of compounds affecting different targets in other apicomplexan parasites, and show that monitoring of TaSP- and Tap104 mRNA levels constitutes a suitable tool for anti-theilerial drug development.

## Highlights

• Quantitative RT real time PCR was used to assess metabolic impairment of *Theileria* schizonts.
• The method was validated with buparvaquone.
• Buparvaquone acts directly and rapidly on the parasite within 1 h of treatment.
• Electron microscopy confirmed these findings.
• A series of anti-parasitic compounds and antibiotics acted primarily on the host cells.

## Graphical abstract

**Figure:**
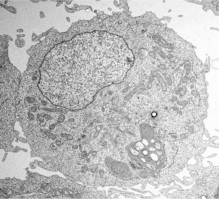

## 1 Introduction

Apicomplexan parasites are responsible for a variety of diseases in humans, pets and/or farm animals, and are thus of considerable medical and economic importance. Those most relevant for farm animals are *Babesia*, *Besnoitia*, *Cryptosporidium*, *Eimeria*, *Neospora*, *Sarcocystis*, *Theileria*, and *Toxoplasma*, causing diseases of great socio-economic impact worldwide. Treatment options for many of these diseases are limited, and include either culling of infected livestock, prevention of infection by vaccination, and/or chemotherapy (Müller and Hemphill, 2013a).

*Theileria* parasitize white blood cells. The most important species are *Theileria parva* and *Theileria annulata*, which cause an acute and usually fatal lymphoproliferative disease in cattle (Morrison and McKeever, 2006). Following transmission by ticks, *T. parva* sporozoites invade lymphocytes, and *T. annulata* infect monocytes, macrophages, dendritic cells and B cells, and, within a few days, develop into schizonts. Development of schizonts causes host cells to undergo proliferation (Dobbelaere et al., 1988). This leads to expansion of the infected cell population and dissemination via the lymphoid system. Cattle in some endemic areas exhibit a degree of innate resistance with low mortality, while cattle introduced from outside succumb to the infection rapidly (Boulter and Hall, 1999).

An important feature of *Theileria* infections is that the host cells are ‘immortalize’ upon infection and resume proliferation (Dobbelaere and Heussler, 1999). Thus, continuous cultures of *T. annulata* could be established by infecting peripheral blood mononuclear cells (PBMCs) with sporozoites (Preston et al., 1998). *T. parva* schizont-infected lymphoblastoid cultures were established from infected animals by employing feeder layer cells and by infecting PBMC with *T. parva* sporozoites (Shkap and Pipano, 2000). These *in vitro* systems are the most important tools to prepare sufficient amounts of the parasite for genome sequencing (Pain et al., 2005) and to study the developmental processes such as invasion of host cells by sporozoites (Shaw, 2003), interactions with the host cell (Heussler and Stanway, 2008) with a focus on the mechanisms of immortalization of the host cell by the parasite (Heussler et al., 2002).

To control bovine theileriosis, three strategies are currently employed: (i) control of the ticks by acaricides, which is expensive, causes environmental damage, and leads to resistance development; (ii) vaccination strategies based on either infection-and-treatment method or attenuated live vaccines, which have been extensively reviewed especially with regard to their risks (McKeever and Morrison, 1998; McKeever, 2007, 2009); and (iii) chemotherapy, which currently represents the most promising anti-theilerial strategy.

Buparvaquone, a hydroxynaphthoquinone related to parvaquone, is the only drug available against *Theileria*, and is a potent selective inhibitor of mitochondrial electron transport, specifically at Complex III (b–c1 complex), in *Theileria*, but also *Plasmodium*, *Eimeria* and *Toxoplasma* (Hudson et al., 1985; Fry and Pudney, 1992). Cattle infected with *T. annulata* or *T. parva* are cured within a few days by a single dose injection (McHardy and Morgan, 1985). The compound has to be administrated, however, during the early stage of infection to avoid the destruction of the immune system that occurs during the advanced stages of the disease. Moreover, first cases of resistance against buparvaquone have been reported (Mhadhbi et al., 2010; Sharifiyazdi et al., 2012). The mode of action and the mechanisms of resistance formation are still unknown. Upon killing the *Theileria* schizont, the infected cell loses its transformed phenotype and rapidly succumbs to apoptosis, making it difficult to distinguish between host cell-mediated effects and the direct action of a given compound upon the parasite (Küenzi et al., 2003).

There is an urgent need for new drugs to treat bovine theileriosis. Before setting up a suitable screening strategy, however, it is paramount to be able to distinguish whether a drug acts primarily against the parasite, or whether it mainly affects the proliferative host cell. The former represents the ideal chemotherapeutic scenario, since the inactivation of the parasite will restore apoptosis of the host cell. In the latter case, chances are high that the drug will affect a host cell target, and adverse side effects are more likely to occur.

In this study we present a quantitative reverse transcriptase real time PCR protocol that allows rapid and reliable monitoring of direct effects upon the *Theileria* metabolism by comparing the mRNA levels of two major schizont-surface proteins, namely TaSP and Tap104 (Witschi et al., 2013; Woods et al., 2013), to host mRNA levels in buparvaquone treated *T. annulata* infected macrophages. Results were confirmed by showing that buparvaquone directly affects the structural integrity of the parasite but not host cell, within few hours of treatment. We subsequently applied this method using a series of compounds affecting different targets in other apicomplexan parasites.

## 2 Materials and methods

### 2.1 Tissue culture media, biochemicals, and drugs

If not otherwise stated, all tissue culture media were purchased from Gibco-BRL (Zürich, Switzerland), and biochemical reagents were from Sigma (St. Louis, MO). Kits for molecular biology were purchased from Qiagen (Hilden, Germany). Buparvaquone (McHardy and Morgan, 1985) was provided by Cross Vetpharm Group Limited (Dublin, Ireland), and was kept as 1.5 mM stock solution in dimethyl sulfoxide (DMSO) at −20 °C. Tetracycline (Sato and Wilson, 2005), clindamycin (Fichera and Roos, 1997), trans-iodo-boranyl-chalcone (TIBC) (Hayashida et al., 2013) and ciprofloxacin (Lizundia et al., 2009) were purchased from Sigma–Aldrich (Buchs, Switzerland). All compounds were kept as 100 mM stock solutions in dimethyl sulfoxide (DMSO) at −20 °C, except ciprofloxacin which was stored in water (pH 4.5) at −20 °C. The dicationic compounds DB745 and DB750 (Schorer et al., 2012), the artemisinine derivatives artemiside and artemisone (Dunay et al., 2009), and the thiazolide nitazoxanide (Esposito et al., 2007b) were stored as above.

### 2.2 Culture of bovine macrophages infected with *T. annulata*

*T. annulata* infected bovine macrophages (TaC12 cells) were cultured as described (von Schubert et al., 2010). For drug treatment experiments, TaC12 cells were seeded into 75 cm^2^ culture flasks, treated with the compound or respective amounts of DMSO as a control and harvested at various time points. Each experimental treatment was carried out in quadruplicate (4 different flasks), and these experiments were repeated at least twice to ensure reproducibility. Cells were detached by removing the medium and resuspending the cell layer in 1 ml of PBS containing 1 mM EDTA. Then, the number of viable cells was counted using a 0.4% trypan blue solution, cells were pelleted by centrifugation (300*g*, 5 min, 4 °C), immediately frozen in liquid nitrogen and stored at −80 °C for subsequent DNA, RNA and protein purification.

### 2.3 Processing of samples for quantitative PCR

Simultaneous DNA, RNA, and protein purification was performed using a Qiagen Allprep DNA/RNA/protein kit according to the standard protocol suitable for cell cultures provided by the manufacturer. DNA was eluted in 100 μl elution buffer from the kit and diluted 20 times in water prior to analysis by quantitative PCR.

RNA was purified including a DNase I digestion (to remove residual genomic DNA) according to the instructions provided by the manufacturer, eluted with 40 μL of RNase-free water, quantified by measuring the absorption at 260 nm, and stored at −80 °C.

### 2.4 Quantitative real-time PCR

Issuing from previously published results (Ros-García et al., 2012), real-time PCR on DNA was performed using the primers Ta18S-F and Ta18S-R (Table 1). As external standards, samples containing between 10 and 10^4^ copies of the plasmid containing the 18S-fragment were included. For this purpose, the 18S fragment was amplified from *T. annulata* genomic DNA and cloned into the vector pCRBluntII (Invitrogen, Carlsbad, Ca) according to the manufacturer’s instructions. Synthesis of cDNA for quantitative RT-PCR was performed with 2 μg of RNA using the Qiagen Omniscript™ kit with random primers according to the manufacturer’s instructions. Quantitative PCR was performed with 10 μL of cDNA (diluted 1:50 in water) using the Quanti TectTM SYBR Green PCR Kit (Roche, Basel, Switzerland) in 20 μL standard reactions containing 0.5 μM of forward and reverse primers (MWG Biotech, Ebersberg, Germany). Furthermore, control PCRs with RNA equivalents from samples that had not been reverse transcribed into cDNA (data not shown) confirmed that no DNA was amplified from any residual genomic DNA that might have resisted DNase I digestion (see above). PCR was started by initiating the ‘Hot-Start’ Taq DNA-polymerase reaction at 95 °C (10 min). Subsequent DNA amplification was performed in 40 cycles including denaturation (94 °C for 15 s), annealing (58 °C for 30 s; 54 °C for actin) and extension (72 °C for 30 s); temperature transition rates in all cycle steps were 20 °C/s. Fluorescence was measured at 76 °C (Ta18S, TaSP, Tap104) or 81 °C (Act) on a Corbett cycler (Corbett Research, Mortlake, Australia). From the quantitative RT-PCR, mean values (±SE) from four biological replicates (independent experiments) were assessed and expression levels were given as values in arbitrary units relative to the amount of actin RNA (Müller et al., 2007; Müller and Hemphill, 2011). Actin was chosen as a transcribed reference gene based on previous results with *Neospora caninum* infected mice (Strohbusch et al., 2008a) and human fibroblasts (Müller and Hemphill, 2011). The results of one out of three independent experiments are shown, all yielding essentially identical results.

### 2.5 Immunofluorescence and immunoblotting

For immunofluorescence, TaC12 cells were seeded on coverslips, treated and harvested as indicated and processed as described previously (von Schubert et al., 2010). The antibodies used in this study are summarized in Table 2.

For immunoblotting, protein pellets obtained from the processing of the samples via the Allprep kit were resuspended in the protein resuspension buffer included in the kit (10^4^ cells/μl), sonicated for 5 s at 20 mA, and heated for 20 min at 60 °C with shaking. The proteins were separated by SDS–PAGE and blotted on nitrocellulose as described (Nillius et al., 2011). The blots were decorated with mouse anti-p104 (1C12) or mouse monoclonal anti-α-tubulin (DM1A) followed by goat-anti-mouse secondary antibodies conjugated to horseradish peroxidase. Chemoluminescence pictures were detected by a CCD (cooled charge coupled devices) camera.

### 2.6 Transmission electron microscopy (TEM)

For TEM, confluent cultures of bovine macrophages infected with *T. annulata* were treated with buparvaquone (150 nM) or with DMSO as a solvent control. Cells were harvested as described above and resuspended in 1 ml ice-cold 100 mM sodium cacodylate buffer pH 7.3, transferred to 1.5 ml Eppendorf tubes, and centrifuged (300*g*, 5 min, 4 °C). Pellets were resuspended in 100 mM cacodylate (pH 7.3) containing 1% glutaraldehyde and fixed overnight at 4 °C. Pellets were then washed three times in 100 mM cacodylate buffer and postfixed in 100 mM cacodylate containing 2% osmium tetroxide for 1 h at room temperature. Pellets were then washed two times in distilled water, contrasted in saturated uranyl acetate for 30 min, dehydrated in an ethanol series (30%, 50%, 70%, 90%, 3 × 100%), and embedded in Epon 820 resin (16). The resin was polymerized at 65 °C over a period of 48 h. Ultrathin sections were cut on a Reichert and Jung ultramicrotome and loaded onto 300-mesh copper grids (Plano GmbH, Marburg, Germany). Staining with uranyl acetate and lead citrate was performed as described previously (Hemphill and Croft, 1997). Finally, grids were viewed on a Phillips 400 transmission electron microscope operating at 60 kV.

### 2.7 Statistics

Statistical analysis of the results was performed with suitable tools from the open source software package R (R Core Team, 2012). Differences exhibiting *p* values of <0.01 were considered significant.

## 3 Results

### 3.1 Assessment of *T. annulata* 18S RNA expression during buparvaquone treatment reveals anti-proliferative effect

**Fig. 1:**
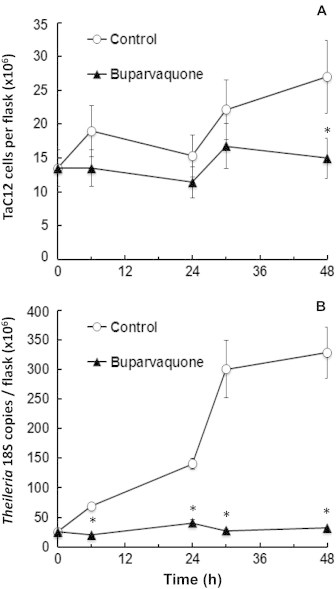
Growth of TaC12 cells in the presence of buparvaquone. 10^7^ cells were seeded at time point 0 in the presence of buparvaquone (150 nM) or equal amounts of DMSO (control). At various time points, cells were harvested, live cells were counted, and in parallel Ta18S-rRNA copy number was quantified by real-time PCR on genomic DNA. Mean values (±SE) for four biological replicates are presented. Values labeled by an asterisk are significantly different (*p* < 0.01) between controls and buparvaquone treated samples. (A) Cell number; (B) 18S copy number.

In order to determine whether the quantitative assessment of RNA expression could be used to investigate drug effects in *T. annulata*, we employed the Ta18S-PCR previously developed for diagnostic purposes (Ros-García et al., 2012). *T. annulata* infected TaC12 cells were cultured in the presence of buparvaquone (150 nM) or the corresponding amount of DMSO as a control. Cells were harvested at various time points (0–48 h), counted, and in parallel the corresponding number of 18S copies was determined by quantitative PCR. Within the time frame of 30 h, the live cell numbers in treated and control cells did not differ substantially, and significant differences in cell numbers were noted earliest after 48 h (Fig. 1A). Conversely, Ta18S RNA expression in control cells started to increase significantly already after 6 h and reached a plateau at 30 h, whereas corresponding expression levels remained at a constantly low level in buparvaquone treated cells (Fig. 1B). Thus, monitoring Ta18S-RNA expression represents a suitable tool to assess the anti-proliferative effects of buparvaquone against *T. annulata* schizonts, and picks up these effects much earlier than monitoring host cell proliferation.

### 3.2 Assessment of mRNA expression coding for the two *T. annulata* schizont proteins TaSP and Tap104 reveals early metabolic impairment of parasites during buparvaquone treatment

**Fig. 2:**
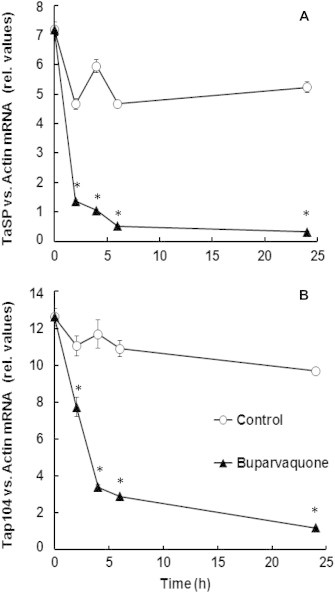
Buparvaquone induces a rapid decrease of *T. annulata* mRNA levels. 10^7^ cells were seeded at time point 0 in the presence of buparvaquone (150 nM) or equal amounts of DMSO (control). At various time points, cells were harvested, RNA was extracted, and mRNA levels of TaSP (A) and Tap104 (B) was quantified by real-time RT-PCR to host-cell actin. Mean values (±SE) for four biological replicates of one representative experiment are presented. All assays were repeated at least twice yielding essentially identical results. Values labeled by an asterisk are significantly different (*p* < 0.01) between controls and buparvaquone treated samples.

**Fig. 3:**
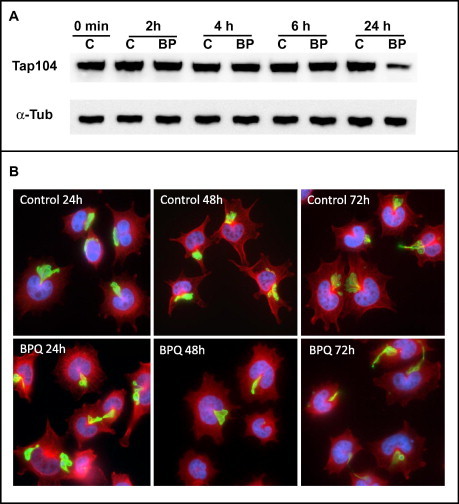
Buparvaquone effects investigated at the protein level. (A) 10^7^ cells were seeded at time point 0 in the presence of buparvaquone (150 nM; BP) or equal amounts of DMSO as a control (C). At various time points as indicated, cells were harvested and processed for SDS–PAGE followed by immunoblotting. Bands corresponding to alpha-tubulin (α-Tub) and to Tap104 are shown. (B) Immunofluorescence showing TaC12 cells cultured in the absence (controls) or presence of buparvaquone for 24, 48 and 72 h, respectively. Cells were stained with antibodies directed against alpha-tubulin (red) and TaSP (green).

TaC12 cells were treated with buparvaquone (150 nM) or DMSO as a control, and were harvested at different time points (2 h, 4 h, 6 h and 24 h). The expression levels of two mRNAs encoding the schizont-specific surface proteins TaSP and Tap104 were determined in relation to host cell actin mRNA by quantitative reverse transcription real time PCR. Upon buparvaquone treatment, the relative TaSP mRNA levels decreased rapidly, within 2–6 h, to background levels (Fig. 2A). Similar results were obtained for Tap104 mRNA (Fig. 2B). As in previous studies (Strohbusch et al., 2008a; Müller and Hemphill, 2011), actin revealed to be a suitable reference. In the time frame shown in Fig. 2, mean actin *C_t_* values were 23.6 with a standard deviation of only 0.2 (data not shown). In parallel, protein samples were collected and immunoblot analysis of Tap104 was performed, while alpha-tubulin was analyzed as a loading control. As shown in Fig. 3A, 24 h of buparvaquone treatment was required before a slight decrease of Tap104 expression was noted. The expression of *Theileria* TaSP and host cell alpha-tubulin during buparvaquone treatment was also investigated by immunofluorescence, over a time frame of 72 h (Fig. 3B). In untreated control cultures all TaC12 cells exhibited an intact microtubular cytoskeleton and, schizonts could be readily visualized in virtually all cells by detection of TaSP during 72 h of culture. In buparvaquone treated TaC12 cells, TaSP staining was still present after 24 h in most cells, and a reduction of TaSP staining intensity could be noted earliest after 48–72 h (Fig. 3B). Immunofluorescence labeling employing an antibody directed against Tap104 yielded essentially identical results. Thus, buparvaquone treatment induces a rapid loss of TaSP- and Tap104-mRNA expression, while on the protein level these two antigens are maintained and still readily detectable for extended periods of time.

### 3.3 Electron microscopy reveals rapid effects of buparvaquone treatment on *Theileria* schizonts within the same time frame as quantitative reverse transcriptase real time PCR

**Fig. 4:**
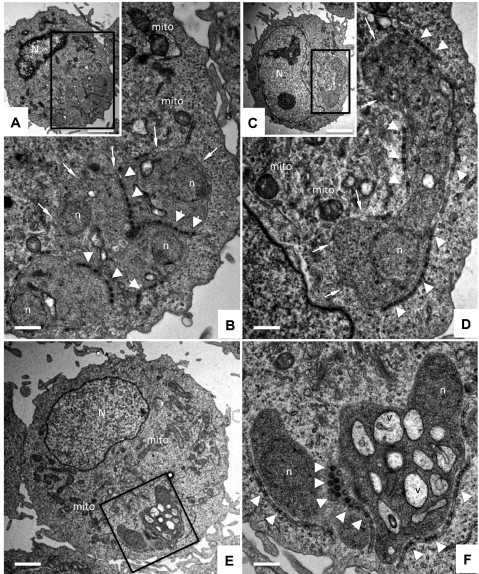
Buparvaquone treatment affects the structural integrity of *T. annulata* schizonts at at early timepoint. (A)–(D) show samples of non-treated control cells fixed and processed at timepoint 0 h (A and B) and 24 h (C and D), with (B) and (D) being higher magnification views of the boxed areas in (A) and (C). (E) Represents a *T. annulata* infected cell treated with buparvaquone for 2 h, and the boxed area is enlarged in (F). *N* = host cell nucleus, mito = host cell mitochondria, *n* = schizont nucleus, white thin arrows point towards the schizont-host cell cytoplasm-interface, white triangles indicate button-like electron-dense structures seemingly acting as membrane connectors. Note the increased vacuolization within the cytoplasm of schizonts in buparvaquone-treated cells (E and F). Bar in *A* = 3.4 μm, *B* = 0.8 μm, *C* = 3.4 μm, *D* = 0.6 μm, *E* = 2 μm, *F* = 0.6 μm.

TaC12 cells treated with buparvaquone (150 nM) or DMSO as a control were harvested at different time points (2 h, 6 h and 24 h) and fixed and processed for TEM analysis. In control cells (Fig. 4A–D) *T. annulata* schizonts were readily detected. In contrast to other apicomplexans, *Theileria* infected host cells lack a parasitophorous vacuole and corresponding membrane, thus schizonts reside freely in the host cell cytoplasm. Typically 2–6 parasite nuclei were visible per section plane. In many instances, abundant numbers of electron dense button-like structures, seemingly acting as membrane connectors and structurally exhibiting some resemblance to tight junctions, were found to be present within individual schizonts, or at their periphery. There were no differences between samples that were fixed and processed just prior to drug treatment (timepoint 0 h, Fig. 4A and B) and those that were processed for TEM after 24 h of culture in the absence of buparvaquone (Fig. 4C and D).

**Fig. 5:**
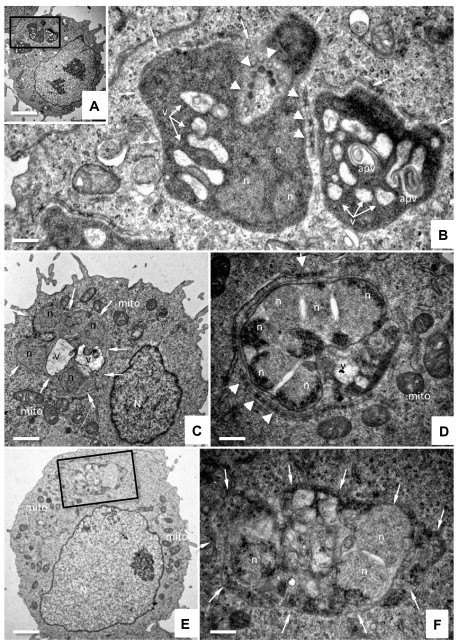
Ultrastructural changes observed after 6 and 24 h of buparvaquone treatment. (A)–(C) show cells processed after 6 h, and (D)–(F) shows specimens processed after 24 h of drug treatment. (A) and (B) (B = higher magnification view of A) show an increased vacuolization (*v*) and accumulation of intravacuolar membrane stacks (apv). In other specimens such as in (C), vacuoles become rather spacious and occupied large parts of the schizont cytoplasm. (D)–(F) show cells processed after 24 h of buparvaquone treatment, with considerable shrinkage of parasite nuclei (*n*) and electron dense deposits within the nuclear matrix and also at the schizont periphery adjacent to the host cell cytoplasm (E) and (F) (at a higher magnification of E). The button-like electron dense structures remained associated with the schizont periphery in some cells (D, marked with white triangles), in others cells (Fig. 5E and F), these structures were completely lost. Note that in all instances the parasite plasma membrane (white arrows) remained intact, and no alterations in the host cell were observed. mito = host cell mitochondria. Bar in *A* = 3.4 μm, *B* = 0.5 μm, *C* = 1 μm, *D* = 0.6 μm, *E* = 1.7 μm, *F* = 0.6 μm.

First ultrastructural changes were evident in *T. annulata* schizonts already after 2 h of drug exposure, and included mainly extensive vacuolization of the schizont cytoplasm. Vacuoles contained amorphous material of varying electron density and unknown nature, or intravacuolar membranous indentions were evident (Fig. 4E and F). The host cell ultrastructure remained unaltered. In many samples fixed after 6 h of buparvaquone treatment (Fig. 5A and B), the degree of cytoplasmic vacuolization of schizonts increased, and accumulation of intravacuolar membrane stacks was often observed. In addition, many button-like electron-dense structures found within *Theileria* schizonts were partially degraded or had lost their characteristic structure. In other specimens, vacuoles became rather spacious and occupied large parts of the schizont cytoplasm (Fig. 5C). After 24 h of buparvaquone treatment, most parasites were heavily damaged. Nuclei exhibited considerable shrinkage, electron dense deposits appeared within the nuclear matrix and also at the schizont periphery adjacent to the host cell cytoplasm (Fig. 5D–F). In some instances, peripheral button-like electron dense structures remained associated with the schizont periphery (Fig. 5D), in other cells (Fig. 5E and F), these structures were completely lost, and the ultrastructural organization of the schizonts was considerably distorted. However, in all instances, the parasite plasma membrane remained morphologically intact and clearly separated the parasite and host cell cytoplasm. Thus, electron microscopy demonstrated the rapid parasite-specific effects of buparvaquone and largely mirrored the results obtained by quantitative TaSP- and Tap104-reverse transcriptase real time PCR.

### 3.4 Assessment of a range of anti-parasitic compounds employing quantitative TaSP and Tap104 reverse transcriptase real time PCR

Monitoring of mRNA expression levels of TaSP and Tap104 was employed to investigate a selection of compounds against *T. annulata* schizonts. Artemisone and artemiside, DB745, DB750, and nitazoxanide have been shown earlier to exhibit *in vitro* activities against other apicomplexan parasites such as *Toxoplasma gondii* and *N. caninum* in the micromolar and submicromolar range (Esposito et al., 2007b; Dunay et al., 2009; Kropf et al., 2012; Mazuz et al., 2012; Schorer et al., 2012), ciprofloxacine and clindamycine target *Plasmodium* and *Theileria* apicoplast constituents such as topoisomerase II and large subunit rRNA, and tetracycline acts on the small subunit rRNA of the plastid (Lizundia et al., 2009). TIBC was reported to markedly inhibit proliferation of *T. parva*-infected lymphocytes by specifically inhibiting MDM2 (Hayashida et al., 2013). The results, summarized in Table 3, show that several compounds had a marked effect on proliferation of host cells after 3 days of culture, such as DB750 and DB745 (used at 1 μM), the two artemisinine derivatives (used at 10 μM), as well as tetracycline and TIBC (at 20 and 10 μM, respectively). However, none of these compounds induced a marked change in the TaSP mRNA expression levels. This indicates that all these compounds, with the exception of buparvaquone, primarily impair host cell metabolism, and do not exert their efficacy by interfering with parasite metabolic activities in the first place.

## 4 Discussion

We here present the development of a sensitive tool, for the assessment of rapid and parasite-specific drug activities in TaC12 cells infected with *T. annulata* schizonts, which is based on the quantitative monitoring of mRNA coding for the two schizont surface proteins TaSP and Tap104 (Seitzer et al., 2007) in relation to host cell actin mRNA expression. Both antigens are also expressed in *T. parva* schizonts (Schnittger et al., 2002), and a similar assay could be potentially applied for that parasite as well. There is a clear need for the identification of novel compounds for the treatment of theileriosis. While buparvaquone is widely used and effective, it has clear limitations such as costs as well as emerging resistance, which require urgent action.

Assessment of the efficacy for drugs in *Theileria* infected cells is not a straightforward matter. *T. parva*-infected lymphocytes and *T. annulata* infected macrophages are transformed and undergo continuous proliferation, and apoptosis of these host cells is restored once the invaders are killed (Dobbelaere and Heussler, 1999). Different assays have been used to investigate the efficacy of anti-theilerial drugs. Since elimination of *Theileria* schizonts will cause host cell apoptosis, it is possible to simply monitor host cell proliferation, either microscopically or by metabolic incorporation of a tracer such as tritiated thymidine (Lizundia et al., 2009). Microscopy offers the added value of visualizing changes in the host cell morphology. Other methods include classical vitality assays 3-(4,5-dimethylthiazol-2-yl)-2,5-diphenyltetrazolium bromide (MTT) or resazurin, but they all have the caveat that not parasite proliferation, but rather host cell proliferation, is quantified (Müller and Hemphill, 2013b).

Most importantly, the assays use to date do not allow to clearly determine whether a compound affects primarily the parasite or the host cell, since accurate controls are missing. Non-infected normal macrophages or B-cells do not naturally proliferate in culture, or only to a limited extent, and are thus not of use to assess potential cytotoxic effects. One possibility is to perform drug assays employing the SV40-transformed cell line of *Theileria* uninfected bovine macrophages (von Schubert et al., 2010) and/or the non-infected B-lymphosarcoma cell line BL3 (Moreau et al., 1999). However, these controls cannot exclude that observed inhibition of a given compound of *Theileria* infected cells is due to impairment of host cell-specific processes instead of parasite-specific inhibition (Lizundia et al., 2009).

Therefore, besides growth or viability assays of treated cells, a marker should be used that allows detecting effects of the compound on the parasite viability in a time window where the host cell is still fully intact. Monitoring of RNA expression thus represents an ideal marker, based on the rationale that intact RNA is only detectable when viable organisms are present in a given tissue or cells, and transcription is depressed once an organism is metabolically impaired. This is confirmed in this study where we showed that the negative impact of buparvaquone on *T. annulata* proliferation can be monitored through assessment of 18S RNA levels in TaC12 cells.

Similar assays, albeit by detecting other RNA targets, have been developed earlier. For instance, one assay based on measuring of mRNA expression levels of dense granule antigen 2, a protein that is expressed in both tachyzoites and bradyzoites of *N. caninum*, has been used to quantify the viability of this parasite within infected host cells, and within infected tissue of experimentally infected animals (Strohbusch et al., 2008a,b; Debache et al., 2009; Lizundia et al., 2009). More recently, van den Bogaart et al. reported on the development and application of a duplex quantitative reverse transcriptase PCR for simultaneous assessment of drug activity against *Leishmania* intracellular amastigotes and their host cells, by quantifying *Leishmania* 18S ribosomal RNA and the human beta-2-microglobulin mRNA (van den Bogaart et al., 2013). In this study, we found that buparvaquone treatment of *T. annulata* infected cells leads to a rapid and specific drop in the expression levels of the two schizont surface antigens TaSP and Tap104 mRNA. This drop is evident already within the first 2–4 h of drug exposure, while TaC12 cells continue proliferation for many more hours before an effect is noted. This drop in metabolic activity of *Theileria* schizonts is clearly mirrored by electron microscopical observations that detect obvious signs of metabolic and structural impairment (vacuolization, autophagy-like features) within the same time window. The progressive deterioration of the schizont ultrastructure within the first 24 h of drug treatment, however, is not readily reflected on the protein level. Alterations in the TaSP and Tap104 expression levels are not detected prior to 24 h, neither by light microscopical inspection, Western blotting nor by immunofluorescence staining, at a timepoint where many parasites have undergone distinct ultrastructural changes. Thus, buparvaquone affects the integrity and thus viability of the *Theileria* schizont long before indirect effects on the host cell due to the onset of apoptosis are visible (Guergnon et al., 2003). Besides the described mode of action involving mitochondrial electron transport (Hudson et al., 1985; Fry and Pudney, 1992), this rapid effect may be also partially due to a reductive activation of the naphthoquinone to the corresponding semiquinone radical directly binding to DNA and thereby inhibiting replication and transcription as proposed for the mode of action of some antitumoral quinones (Butler and Hoey, 1987).

Subsequently, we investigated a number of compounds which have been shown earlier to display pronounced activities against other apicomplexan parasites. Some of these induced a substantial depression in cell proliferation within 3 days of culture. The artemisinine derivatives artemisone and artemiside exhibited interesting *in vitro* activities against *T. gondii* tachyzoites, and were also proven to act against experimentally induced acute and reactivated toxoplasmosis in mice (Dunay et al., 2009). Artemisone was also found to protect gerbils against experimental *N. caninum* infection (Mazuz et al., 2012). The nitro-thiazolide nitazoxanide exhibited pronounced *in vitro* activity against *N. caninum* (Esposito et al., 2007a,b; Mazuz et al., 2012). Interestingly, nitazoxanide was also found to be highly active against cancer cells such as the colon carcinoma cell line Caco2, and this effect was shown to be based on the interaction of the compound with glutathione-S-transferase Pi (GSTP1), which is highly expressed in colon cancer cells (Müller et al., 2008). Thiazolide-induced apoptosis in colorectal cancer cells is mediated via the Jun kinase-Bim axis and it would be interesting to investigate whether this also is relevant in *Theileria*-infected cells. Pentamide derivatives, such as the two closely related dicationic compounds DB750 and DB745, previously shown to be highly effective at 1 μM against *N. caninum* tachyzoites *in vitro* and also efficacious in the experimental mouse model (Debache et al., 2011; Schorer et al., 2012), did also have a significant impact on TaC12 proliferation. However, as evidenced by quantitative reverse transcriptase PCR, none of these compounds was primarily targeting the parasite metabolism, since TaSP mRNA expression levels were not notably altered after 24 h. Of those compounds that have been postulated to target the apicoplast, only tetracycline (at 20 μM) showed a marked effect, but again, this was not associated with a primary action on the parasite metabolism. In contrast to studies on *T. parva* (Lizundia et al., 2009), ciprofloxacin did not negatively affect neither *T. annulata* schizont mRNA expression nor the proliferation of *T. annulata*-infected cells. However, our investigations on the effects of ciprofloxacin treatment of *T. parva* infected lymphocytes confirmed the findings obtained by Lizundia et al. (data not shown), demonstrating that there must be some substantial differences between *T. parva* and *T. annulata*. However, the question whether these differences are related to the host cell or are based on different metabolic requirements of the two parasites needs to be addressed. Finally, TIBC applied at 10 μM was also shown to have a profound impact on TaC12 proliferation, but parasite mRNA expression was not affected, confirming earlier results that have shown that TIBC interacts with host MDM2 (Hayashida et al., 2013).

Our results suggest the following strategy for the identification of novel anti-theilerial compounds. In as first round, a series of compounds is tested on TaC12 cells determining growth or a viability parameter (e.g. via a resazurin assay) after a couple of days. Compounds inhibiting growth are then tested in a second round on TaC12 cells during 24 h. Then RNA is extracted and TaSP vs. actin levels are determined. A compound as effective as buparvaquone (or better) would reduce relative mRNA levels to background values whereas compounds affecting the host cell would reduce actin levels to similar extents or even faster than TaSP levels.

Taken together, the method described here does not reveal the mechanism of action of a compound, but constitutes a promising tool in the further assessment of drugs for the chemotherapy of bovine theileriosis.

## Conflict of interest

The authors declared that there is no conflict of interest.

## Figures and Tables

**Table 1:** Overview of primers used in this study.

Gene	Accession-no.	Primer	Sequence
Ta18S	KF429795.1	Ta18S_F	GACCTTAACCTGCTAAATAGG
		Ta18S_R	CAGGCCTCTCGGCCAAGG
Tap104	TA08425	Tap104_F	TCATAGGTCTACAGAACTGGA
		Tap104_R	TTTAGGTGGTTCTGGACCCT
TaSP	TA17315	TaSP_F	AGCAGCCCCTTGTCATGGG
		TaSP_R	TAATAGCTTTTGCACGGAGGA
Actin	NM_001100	α-AC1	GAGACCACCTACAACAGCATCATG
		α-AC2	CACCTTGATCTTCATGGTGCTGGG

**Table 2:** Overview of antibodies used in this study.

Antibody	Target	Dilution	Source
Rabbit anti-TaSP	*T. annulata* schizont surface protein TaSP	1:1000 IF	Isabel Roditi, University of Bern, Switzerland
Mouse 1C12	*T. annulata* schizont surface protein p104	1:200 IF	Brian Shiels, University of Glasgow, United Kingdom
		1:250 WB	
Mouse DM1A	Alpha-tubulin	1:5000 IF	SIGMA
		1:5000 WB	
Goat-anti-rabbit Alexa fluor 488	Rabbit IgG	1:1000 IF	Molecular probes
Goat-anti-mouse Texas red	Mouse IgG	1:1000 IF	Molecular probes
Rabbit-anti-mouse-HRP	Mouse IgG	1:2000 WB	DAKO

IF, immunofluorescence; WB, Western blot.

**Table 3:** Efficacy test against TaC12 cells employing several compounds known to be effective against various apicomplexan parasites.

Compound	Concentration	Viable TaC12 (×10^5^)	TaSP vs. actin
Control	0.1%	32.0 ± 2.2	3.19 ± 0.10
Buparvaquone	150 nM	8.8 ± 1.8^∗^	0.24 ± 0.01^∗^
Ciprofloxacin	10 μM	33.4 ± 3.0	2.34 ± 0.36
Clindamycine	10 μM	34.0 ± 1.2	2.97 ± 0.09
Tetracycline	20 μM	11.4 ± 3.4^∗^	2.83 ± 0.45
Artemiside	10 μM	2.2 ± 0.2^∗^	5.04 ± 0.21
Artemisone	10 μM	4.6 ± 0.7^∗^	3.39 ± 0.53
Nitazoxanide	10 μM	22.8 ± 3.1	2.64 ± 0.08
DB745	1 μM	0 ± 0^∗^	2.86 ± 0.46
DB750	1 μM	4.2 ± 1.3^∗^	1.72 ± 0.23
TIBC	10 μM	9.0 ± 3.0^∗^	2.70 ± 0.43

TaC12 cells were treated with various compounds or with DMSO as a solvent control as indicated. Viable cells were counted in the presence of Trypan blue after 3 d or harvested for RNA extraction after 24 h. Mean values (±SE) for four biological replicates of one representative experiment are presented. All assays were repeated at least twice yielding essentially identical results. Values labeled by an asterisk are significantly different (*t*-test; *p* < 0.001) from the control.
